# Ultrastructural Signs of High Functional Activity of Neuromuscular Synapses in Aging Rats After Photobiomodulation

**DOI:** 10.3390/cells15080710

**Published:** 2026-04-17

**Authors:** Tatyana Vasyagina, Daria Nefedova, Andrey Seliverstov, Natalya Shchelchkova, Marina Bugrova, Anna Bavrina

**Affiliations:** 1 Federal State Budgetary Educational Institution of Higher Education Privolzhsky Research Medical University of the Ministry of Health of the Russian Federation, 603005 Nizhny Novgorod, Russia; nifka22@mail.ru (D.N.); andselisk@gmail.com (A.S.); n.shchelchkova@mail.ru (N.S.); marysmir@mail.ru (M.B.); annabavr@gmail.com (A.B.); 2 Institute of Biology and Biomedicine, Lobachevsky State University of Nizhny Novgorod, 23 Gagarina Ave., 602022 Nizhny Novgorod, Russia

**Keywords:** skeletal muscle, neuromuscular junction, regeneration, aging, photobiomodulation, transmission electron microscopy

## Abstract

**Highlights:**

**What are the main findings?**

- Light and transmission electron microscopy revealed characteristic age-related changes in muscle fibers and neuromuscular synapses.
- Photobiomodulation causes noticeable ultrastructural reorganization of neuromuscular junctions in aged rats, including an increase in the number of active zones, elongation of the postsynaptic membrane, narrowing of the synaptic cleft, and mitochondrial hyperplasia.

**What are the implications of the main findings?**

- Photobiomodulation promotes compensatory structural reorganization of neuromuscular junctions and enhances their functional activity.
- These results highlight the therapeutic potential of photobiomodulation for alleviating age-related sarcopenia and muscle weakness.

**Abstract:**

Aging is characterized by progressive degeneration of neuromuscular junctions (NMJs), which significantly contributes to muscle weakness and the development of sarcopenia. Photobiomodulation (PBM), a non-invasive therapeutic method based on the use of low-intensity light, has shown promising results in mitigating muscle degeneration in both experimental and clinical studies. The aim of this study was to evaluate the ultrastructural effects of photobiomodulation on neuromuscular junctions and skeletal muscle fibers in the *m. vastus lateralis* muscle of aged rats using light and transmission electron microscopy. Male Wistar rats (18 months old, body weight 650–800 g, *n* = 10) were subjected to photobiomodulation of the right *m. vastus lateralis* muscle (650 nm, 6 J/cm^2^, four consecutive daily sessions of 3 min each). The contralateral left limb served as an untreated control. Muscle samples were analyzed by light and transmission electron microscopy. Histological examination revealed typical age-related changes in control muscles, including variability in muscle fiber diameter, centrally located nuclei, and an increased volume of connective tissue. Ultrastructural analysis confirmed signs of skeletal muscle aging, such as myofibril fragmentation, sarcomere disorganization, lipofuscin accumulation, and tubular aggregate formation. Morphometric analysis of neuromuscular junctions after photobiomodulation showed an increase in the number of active zones on the presynaptic membrane, elongation of the postsynaptic membrane, and a reduction in the width of the synaptic cleft. In addition, mitochondrial hyperplasia was observed in presynaptic terminals, while the total number of synaptic vesicles decreased. These findings indicate a compensatory reorganization of neuromuscular junctions and suggest that photobiomodulation can enhance their functional activity in aged skeletal muscle.

## 1. Introduction

Skeletal muscles, which make up approximately 40% of human body mass, are highly organized structures responsible for a wide range of functions, including locomotion, maintenance of postural stability, and performance of complex motor tasks [1]. This tissue is characterized by pronounced plasticity, enabling it to adapt to various physiological and pathological conditions through remodeling of the myofibrillar apparatus and metabolic pathways [2,3].

The neuromuscular junction (NMJ) is a key structure that ensures efficient signal transmission from α-motor neurons to muscle fibers. This process is mediated by the release of acetylcholine into the synaptic cleft, which triggers sarcolemmal depolarization, calcium release from the sarcoplasmic reticulum, and subsequent myofibril contraction [4,5].

With advancing age, progressive degenerative changes occur in both the nervous and muscular systems, significantly contributing to denervation, muscle weakness, and sarcopenia [6,7]. Although NMJs remain relatively stable throughout most of adult life, they undergo substantial ultrastructural alterations during aging, including simplification of postsynaptic folds, a reduced number of active zones, and mitochondrial dysfunction [8].

Additional hallmarks of muscle aging include a decreased cross-sectional area of muscle fibers, lipofuscin accumulation, increased connective tissue content, impaired mitochondrial function, reduced calcium storage capacity of the sarcoplasmic reticulum, loss of satellite cells, and the formation of tubular aggregates [9,10,11]. Recent studies have also highlighted the contribution of elevated oxidative stress to neuromuscular junction degeneration and the progression of muscle weakness [12].

Photobiomodulation (PBM) is a non-invasive therapeutic approach that uses low-intensity red and near-infrared light to stimulate cellular functions. Its primary mechanism involves photon absorption by cytochrome c oxidase (Complex IV) in the mitochondrial respiratory chain, leading to photodissociation of inhibitory nitric oxide, restoration of electron transport, increased mitochondrial membrane potential, and enhanced ATP synthesis [13]. In aged skeletal muscle, these effects are accompanied by reduced oxidative stress and inflammation, as well as activation of satellite cell proliferation and muscle fiber regeneration [14,15]. Of particular relevance to the present study, PBM has been shown to exert beneficial effects at the neuromuscular junction level by improving mitochondrial function in presynaptic terminals, increasing the number of active zones, enhancing synaptic vesicle turnover, and promoting postsynaptic membrane stabilization [16].

The aim of this study was to evaluate the ultrastructural effects of photobiomodulation on neuromuscular junctions and skeletal muscle fibers in the *m. vastus lateralis* muscle of aged rats using light and transmission electron microscopy.

## 2. Materials and Methods

The study was conducted on ten male Wistar rats (18 months old, body weight 650–800 g) obtained from the breeding colony of the Federal State Budgetary Educational Institution of Higher Education “Privolzhsky Research Medical University” of the Ministry of Health of the Russian Federation. The animals were housed in groups of 3–4 per standard polycarbonate cage under controlled environmental conditions (temperature 22 ± 2 °C, relative humidity 60 ± 5%, 12:12 h light–dark cycle) with *ad libitum* access to standard rodent chow and filtered tap water. To minimize stress, the cages were enriched with nesting material and shelter tubes. All procedures were performed in a dedicated procedure room within the animal facility.

Photobiomodulation sessions were non-invasive. Treatment consisted of four consecutive daily sessions of 3 min each, conducted between 09:00 and 11:00 h to avoid the influence of circadian rhythms. No expected or unexpected adverse effects were observed during the study. All ten animals tolerated the procedure well, with no signs of skin irritation, behavioral changes, weight loss, or any other adverse reactions. Immediately after each session, the animals were returned to their home cages and closely monitored. All staff involved in animal care were trained and certified in laboratory animal science. Daily clinical monitoring revealed no deviations from normal grooming, locomotor activity, food intake, or social behavior. All animals completed the study, and none were excluded during either the experimental procedure or data analysis.

The study was conducted in accordance with the “Rules for the Care and Use of Laboratory Animals” (Russia, 2010) and strictly adhered to the ethical principles of the European Convention for the Protection of Vertebrate Animals used for Experimental and Other Scientific Purposes (Strasbourg, 1986; ETS No. 123).

**Figure 1:**
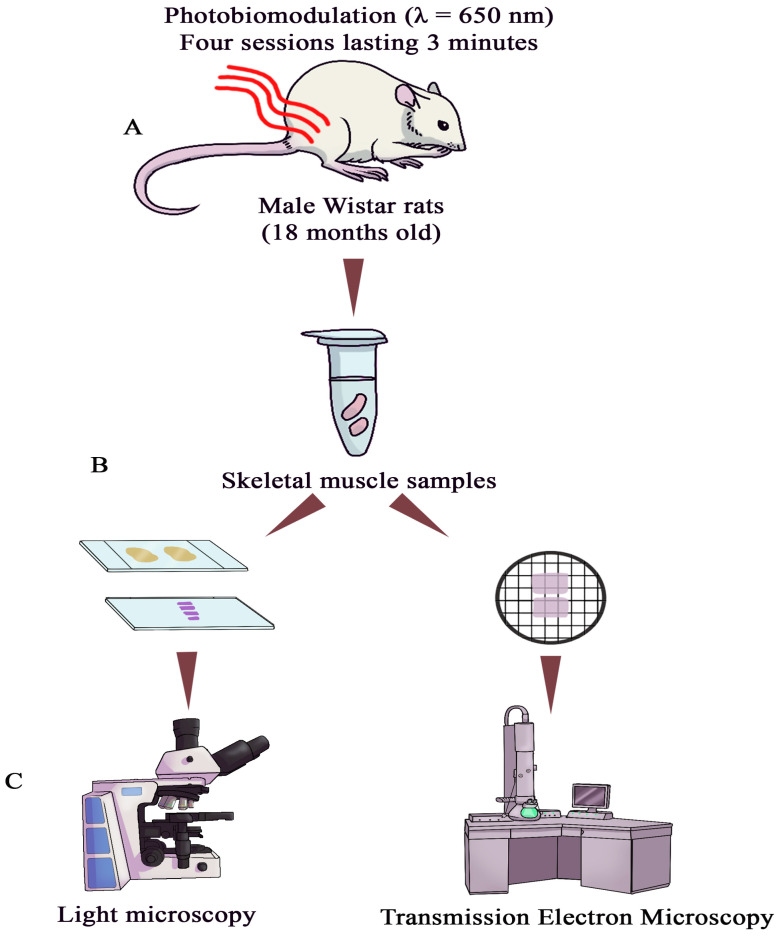
Experimental design of the study. (**A**) Male Wistar rats (*n* = 10; age 18 months; body weight 650–800 g). Photobiomodulation (PBM) was applied to the right *m. vastus lateralis* muscle of the thigh in four consecutive daily sessions of 3 min each (wavelength 650 nm; power density 50 mW/cm^2^). The contralateral left limb served as an untreated control. (**B**) Muscle tissue samples were collected 1 day after the final PBM session. (**C**) Samples were processed for light and transmission electron microscopy.

The object of the study was the right *m. vastus lateralis* muscle, a knee extensor (Figure 1A). To minimize potential confounding factors, a contralateral paired design was employed, in which each animal served as its own control: the right *m. vastus lateralis* muscle received photobiomodulation, while the left muscle remained untreated. This approach effectively controlled for inter-individual variability related to age, genetics, and systemic factors. All samples for morphological analysis were coded, and the investigator performing the light and electron microscopy evaluation was blinded to group allocation. All other procedural variables were strictly standardized across animals.

### 2.1. Photobiomodulation Mode

Photobiomodulation was applied to the right *m. vastus lateralis* muscle of 18-month-old male Wistar rats using a Spectr LC-02 laser diode physiotherapy device (model 02; ITMO Laser Center, St. Petersburg, Russia). Treatment was performed once daily for four consecutive days. Each session lasted 3 min. The irradiation parameters were as follows: wavelength 650 ± 30 nm (red LED), spot area 0.002 m^2^, power density 50 mW/cm^2^ (continuous wave mode), and energy density 6 J/cm^2^ per session.

These parameters (650 nm, 50 mW/cm^2^, 6 J/cm^2^ per session, four consecutive daily sessions) were selected based on previously published protocols shown to effectively stimulate mitochondrial function in skeletal muscle without causing thermal damage [17,18].

Red light in the 630–680 nm range is well absorbed by cytochrome c oxidase (Complex IV of the mitochondrial respiratory chain) and provides an optimal balance between tissue penetration depth and photobiological efficacy. The wavelength of 650 nm is among the most extensively studied and effective for stimulating skeletal muscle tissue. Energy density is a critical therapeutic parameter. A fluence of 6 J/cm^2^ is considered an average therapeutic dose (“the golden mean”). Doses below 3 J/cm^2^ are often insufficient to elicit a biological response, whereas doses above 10–12 J/cm^2^ may induce an inhibitory effect due to the biphasic dose–response relationship of photobiomodulation. For skeletal muscle and neuromuscular junctions, an energy density of 4–8 J/cm^2^ is regarded as optimal for enhancing mitochondrial activity without causing thermal damage. Each session lasted 3 min, which ensures a comfortable and safe exposure duration. Longer irradiation times (>5–7 min) may lead to undesirable thermal effects. The short 4-day treatment course is typical for animal studies aiming to evaluate acute and subacute morphological changes and was chosen to assess early regenerative responses 24 h after the final session. The *vastus lateralis* muscle was selected as the target tissue because it is a large mixed muscle containing both slow- and fast-twitch fibers and is highly susceptible to age-related atrophy in both rats and humans. The contralateral paired design allowed us to clearly distinguish the local effects of photobiomodulation from potential systemic influences.

To ensure rapid tissue collection without chemical exposure, the animals were anesthetized using gas anesthesia with the EZ-AF9000 Auto Flow System (E-Z Systems Inc., Palisades Park, NJ, USA) and isoflurane (3–4% in oxygen at a flow rate of 1 L/min for 1 min). Decapitation was performed using a rat guillotine (AE 1601; Open Science, Moscow, Russia).

### 2.2. Light Microscopy

The tissue samples were fixed in 10% neutral buffered formalin (cat. no. R-001/5000; Labiko, Moscow, Russia) for 48 h at room temperature. After fixation, the samples were dehydrated in a graded series of ethanol solutions (50%, 60%, 70%, 80%, 95%, and 95%, 1 h each), with an additional overnight incubation in 95% ethanol. The samples were then cleared in chloroform (cat. no. 210100; LenReactiv, St. Petersburg, Russia) twice for 40 min at 37 °C using a TS-1/80 SPU thermostat (cat. no. 1001; Smolensk SKTB SPU, Smolensk, Russia). Subsequently, the tissues were impregnated in a 1:1 mixture of chloroform and paraffin for 1.5 h at 37 °C, embedded in paraffin (cat. no. 10342; BioVitrum, St. Petersburg, Russia), and incubated at 58 °C for 24 h to complete the embedding process. Serial sections 5–7 μm thick were cut from the paraffin blocks using a Leica RM 2245 microtome (Leica Biosystems, Wetzlar, Germany) and mounted onto microscope slides (cat. no. 0303-2125; BioVitrum, St. Petersburg, Russia).

Van Gieson staining was performed on paraffin sections to visualize connective tissue components (Figure 1B). Sections were deparaffinized in ortho-xylene (Labiko, cat. no. R-021/1000, Moscow, Russia) twice for 2 min each, rehydrated in a descending ethanol series (95%, 95%, 95%, and 70%, 2 min each), and washed in distilled water for 2 min. Nuclei were stained with Weigert’s iron hematoxylin (BioVitrum, cat. no. HK-VG-AQC3, St. Petersburg, Russia) for 10 min, followed by rinsing in warm tap water for 10 min. Connective tissue was counterstained with picrofuchsin Y (BioVitrum, cat. no. HK-VG-AQC3, St. Petersburg, Russia) for 5 min. The sections were then dehydrated in an ascending ethanol series (70%, 95%, and 95%, 1 min each), cleared in ortho-xylene (3 × 1 min), and mounted with Glasseal mounting medium (Labiko, cat. no. R-028/500, Moscow, Russia) under 24 × 50 mm coverslips (MiniLab, cat. no. 12003322, Moscow, Russia).

Morphological evaluation was performed using a BioOptic S-400 bright-field microscope (BioVitrum LLC, St. Petersburg, Russia) equipped with a trinocular head and an AmScope MU633-BI digital camera (6.3 MP, CMOS BSI sensor; AmScope, Irvine, CA, USA). Images were acquired at 20× and 40× magnification using plan achromatic objectives. Image processing and morphometric analysis were carried out using AmScope software (version 4.11; AmScope, Irvine, CA, USA) and Fiji (ImageJ version 1.54p; National Institutes of Health, Bethesda, MD, USA).

### 2.3. Transmission Electron Microscopy (TEM)

Muscle tissue samples were fixed at room temperature in 2.5% glutaraldehyde (cat. no. 340855; Fluka, Sigma-Aldrich, Buchs, Switzerland) and post-fixed in 1% osmium tetroxide (cat. no. 19100; Electron Microscopy Sciences, Hatfield, PA, USA), both prepared in 0.1 M phosphate buffer, pH 7.4 (cat. no. P4417; Sigma-Aldrich, St. Louis, MO, USA).

The fixation time was 1 h for each step. Between fixation steps, the samples were washed in the same 0.1 M phosphate buffer for 10 min. Dehydration was performed in a graded series of ethanol solutions: 50% (3 × 5 min), 70% (10 min), 80% (10 min), 95% (3 × 10 min), and 100% (2 × 10 min). At the 70% ethanol stage, en bloc staining was carried out with 1% uranyl acetate (cat. no. 02624-AB; SPI-Chem, Structure Probe, Inc., West Chester, PA, USA) in 70% ethanol at 4 °C for 12 h. After dehydration, the samples were transferred to a 1:1 mixture of 100% ethanol and 100% acetone (cat. no. 3809.1000; Komponent-Reactiv, Moscow, Russia) for 15 min, followed by two changes of 100% acetone (10 min each).

Impregnation with a mixture of epoxy resins (Epon 812, Araldite M, and DDSA in a ratio of 1:1:2.61 [*v*/*v*]) was performed as follows: —1:1 mixture of epoxy resins and acetone for 1 h; —2:1 mixture of epoxy resins and acetone for 12 h; —pure epoxy resin mixture for 1 h.

Subsequently, the samples were embedded in a complete mixture of Epon 812 (cat. no. 45345; Fluka, Sigma-Aldrich, Buchs, Switzerland), Araldite M (cat. no. A3183; Sigma-Aldrich, St. Louis, MO, USA), DDSA (cat. no. 45346; Sigma-Aldrich, St. Louis, MO, USA), and DMP-30 (cat. no. 13600; Electron Microscopy Sciences, Hatfield, PA, USA) in a ratio of 1:1:2.61:0.06 (*v*/*v*). Polymerization was carried out with a gradual increase in temperature: 37 °C for 12 h, 45 °C for 48 h, and 60 °C for 1 h.

**Figure 2:**
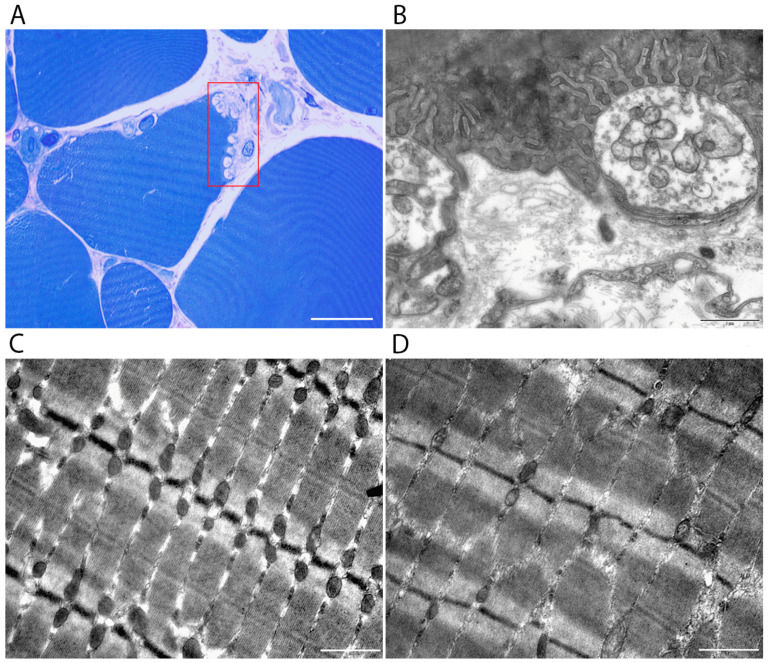
Stages of morphological analysis and representative structures in the rat vastus lateralis muscle. (**A**) Semithin section stained with methylene blue and basic fuchsin, showing neuromuscular junctions (outlined in red). (**B**) Transmission electron micrograph of a neuromuscular junction. (**C**) Type I (slow oxidative) muscle fiber containing numerous mitochondria and narrow, loosely packed myofibrils. (**D**) Type II (fast glycolytic) muscle fiber containing few mitochondria and wide, densely packed myofibrils. Scale bars: 15 µm (**A**); 1 µm (**B**–**D**).

Semithin (0.5–1 μm) and ultrathin (60–80 nm) sections were cut using a Leica UC7 ultramicrotome (Leica, Vienna, Austria) (Figure 1C). Serial semithin sections were stained with methylene blue, azure II, and basic fuchsin according to Humphrey and Pittman [19] to identify the localization of neuromuscular junctions (Figure 2A).

Ultrathin sections were contrasted with a 1% aqueous solution of uranyl acetate and lead citrate (Cat. no. 02530-AB, SPI-Chem, West Chester, PA, USA) according to Venable and Coggeshall [20], and used for morphometric analysis (Figure 2B). Muscle tissue was examined with a Morgagni 268D transmission electron microscope (FEI Company, Eindhoven, Netherlands) at an accelerating voltage of 80 kV. The working magnification ranged from 4500× to 36,000×.

### 2.4. Morphometric Analysis of Nerve Terminal

Morphometric analysis of neuromuscular junctions allows a quantitative assessment of structural changes that reflect synaptic functional efficiency. The ultrastructural features of the NMJ were evaluated using ImageJ software, version 2.14.0, before and after PBM in 40 samples with nerve ending cross-sectional areas ranging from 1.5 to 7.4 μm^2^ [21]. Samples with a small area (<1 μm^2^) that did not contain mitochondria were excluded from the study. The following key parameters were selected for stereological analysis of presynaptic terminals: the relative volumes of mitochondria, synaptic vesicles, and axoplasm, which reflect the state of energy metabolism, mediator storage, and signal transmission stability (Table A1, Appendix A).

The relative volume of the axoplasm (including endosomes and elements of the endoplasmic reticulum) and organelles in nerve endings was determined using the planimetric method. The determination of the relative volume of any structure (Vvi)—the total volume of this structure (Vi) within a unit volume of the nerve ending (Vt)—is based on Delesse’s principle, according to which Vvi = Aai, where Aai is the area occupied by structure i on the cross-sectional area [22].

The parameters of the neuromuscular junction were as follows: cross-sectional area and perimeter of the presynaptic terminal, its major and minor diameters (DMAX and DMIN, respectively), and their ratio. The lengths of the presynaptic and postsynaptic membranes (LPRE and LPOST), the width of the synaptic cleft (WSC), and the number of active zones (NAZ) were measured. Two coefficients were then calculated: PPT/LPOST (perimeter of the nerve ending to the length of the postsynaptic membrane) and LPRE/LPOST (length of the presynaptic to the length of the postsynaptic membrane). The condition of mitochondria was assessed by the number of mitochondrial profiles per 10 μm^2^ of the nerve ending area and by the ratio of normal to defective mitochondria (Table A1, Appendix A).

In the neuromuscular junction, synaptic vesicles containing neurotransmitters are organized into three main functional pools that differ in their localization, readiness for exocytosis, and role in signal transmission [23,24,25]:

1. The readily releasable pool (RRP) is a small group of vesicles located directly adjacent to the presynaptic membrane in the active zone of the synapse. The vesicles in this pool are “loaded” and ready for immediate exocytosis upon the arrival of an action potential. They participate in rapid synaptic transmission, with release occurring within milliseconds after depolarization. After release, they are quickly replenished from other pools.
2. The reserve pool (RP) consists of a large number of vesicles located in the cytoplasm of the presynaptic terminal, away from the active zone. These vesicles do not participate in immediate transmission but serve as a reserve during prolonged or intense stimulation. During sustained activity, vesicles from the reserve pool are mobilized and transported to the active zone via cytoskeletal elements. Their mobilization is regulated by calcium and several proteins, such as synapsin.
3. The recycling pool (RPc) is intermediate in both function and localization between the RRP and RP. Vesicles in this pool are situated closer to the active zone than those in the reserve pool but are not as tightly associated with it as in the RRP. They participate in maintaining synaptic transmission at moderate stimulation frequencies and continuously replenish the RRP through endocytosis and vesicle reformation after exocytosis. This pool ensures the stability of synaptic transmission during repeated action potentials.

The three pools work in concert to ensure both a rapid response to a single signal and stable transmission during prolonged neuronal activity. Based on this classification, only docked vesicles in contact with the presynaptic membrane (≤50 nm, in a single row along the active zone) were assigned to the RRP during morphometric evaluation. The RPc vesicles were counted within the recycling zone at a distance of 50–300 nm from the active zone, while the RP included vesicles located more than 300 nm from the active zone.

### 2.5. Statistical Analysis

Statistical analysis was performed using IBM SPSS Statistics (version 27). The normality of data distribution was assessed using the Shapiro–Wilk test due to the small sample size. Because the data were not normally distributed, the nonparametric Mann–Whitney U test was used to identify significant differences between groups. The results are presented as the median [25th–75th percentile]. Statistical significance was set at *p* ≤ 0.05. The percentage change in quantitative data was calculated relative to the median using the formula: (median_after − median_before)/median_before × 100%.

## 3. Results

### 3.1. Morphological and Ultrastructural Characteristics of Skeletal Muscle in Aging Rats Before and After PBM

**Figure 3:**
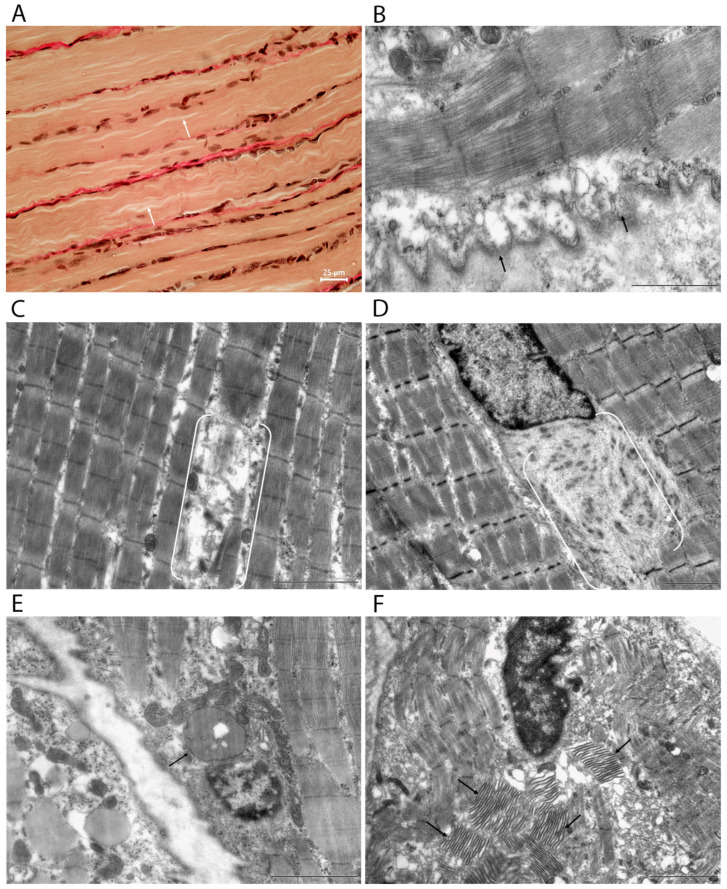
Aging features in control left vastus lateralis muscle from aged rats at the tissue and ultrastructural levels. (**A**) Light microscopy (van Gieson stain) showing variability in muscle fiber diameter within a fascicle, the presence of fibers with longitudinal cleavage (arrows), and an increase in the volume of interstitial connective tissue. (**B**–**F**) Transmission electron microscopy images. (**B**) Sarcolemmal twisting (arrow) and selective myofilament degradation. (**C**) Myofiber fragmentation (double staples). (**D**) Sarcomere disorganization (double staples). (**E**) Large autophagolysosomes in the perinuclear region (arrow). (**F**) Tubular aggregates within the muscle fiber (arrows). Scale bars: 25 μm (**A**); 1 μm (**B**); 2 µm (**C**–**F**).

Histological sections of skeletal muscle stained using the Van Gieson method demonstrated the following signs of aging: noticeable variability in muscle fiber diameter within a single bundle, the presence of fibers showing longitudinal splitting, and an increased volume of interstitial connective tissue (Figure 3A).

Ultrastructural analysis of the samples revealed different types of muscle fibers (Figure 2C,D). Some fibers were type I (slow oxidative), characterized by numerous mitochondria and narrow, loosely packed myofibrils. Others were type II fibers (fast glycolytic), containing fewer mitochondria and wide, tightly packed myofibrils. In the *m. vastus lateralis* of old rats, fast type II fibers (mainly IIA and IIB) predominated, accounting for approximately 60–80% of the total fiber population.

Regardless of fiber type, myofibrillar fragmentation with breaks in the Z-line region is often observed, along with sarcolemmal tortuosity and selective degradation of myofilaments. There is also disruption of the regular packing of sarcomeres, including blurring of the boundaries between the I and A bands and local loss of transverse striations (Figure 3B–D). In addition, large autophagolysosomes and lipofuscin granules accumulate in the perinuclear zone (Figure 3E). Tubular aggregates are frequently found—rounded or oval structures consisting of orderly arranged membrane tubules with diameters of 50–80 nm, localized in the subsarcolemmal and intermyofibrillar regions (Figure 3F).

**Figure 4:**
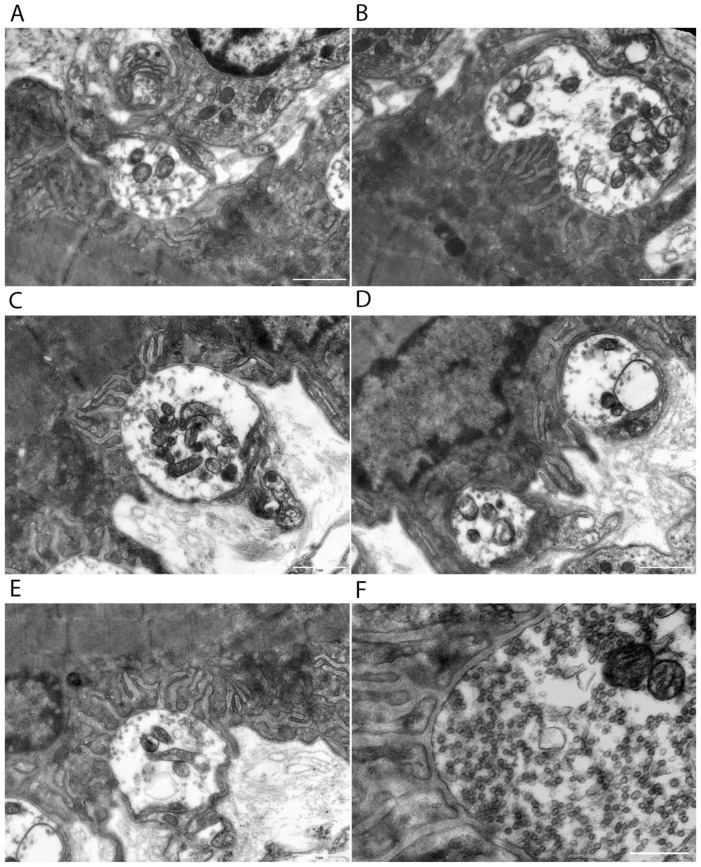
Ultrastructural features of presynaptic terminals in control left vastus lateralis muscle of aged rats. (**A**–**F**) Electron micrographs. (**A**–**E**) Variation in shape and size of axon terminals innervating a single muscle fiber. (**F**) Presynaptic terminal containing synaptic vesicles organized into a reserve pool (RP), a recycling pool (RPc), and a pool ready for release (RRP), located near the active zone of the presynaptic membrane (explained in text). Scale bars: 1 μm (**A**–**E**); 0.5 μm (**F**).

Neuromuscular junctions exhibit marked morphological heterogeneity (Figure 4A–E). The shape of the presynaptic terminals varies from round to oval, and their sizes differ within a single preparation. Within the nerve endings, synaptic vesicles show an uneven distribution, ranging from moderately dense to markedly sparse packing (Figure 4F).

Mitochondria are present in varying numbers, ranging from one or two to ten or more. Some display an electron-dense matrix with well-preserved cristae, while others show fragmented cristae or signs of swelling. A Schwann cell surrounds the vast majority of presynaptic terminals. The degree of contact with glial elements varies, from dense, nearly complete coverage of the terminals to localized, point-like interactions.

**Figure 5:**
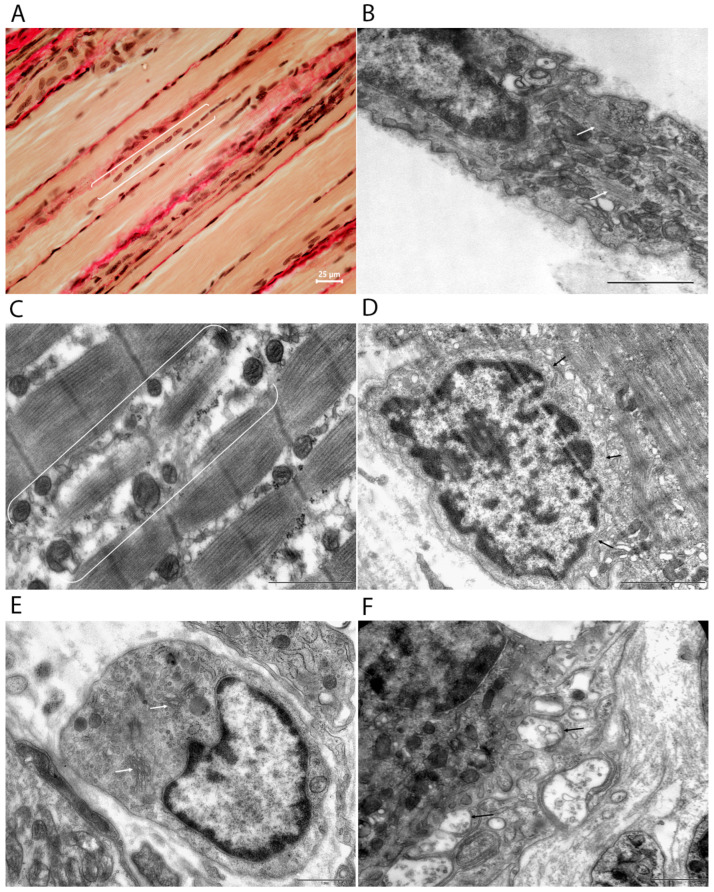
Morphological and ultrastructural signs of fiber regeneration in the right vastus lateralis muscle of the thigh in old rats after photobiomodulation. (**A**) Light micrograph (Van Gieson staining) showing chains of myoblast nuclei aligned between mature muscle fibers. (**B**–**F**) Transmission electron micrographs. (**B**) Fragment of a myoblast containing scattered myofibrils (arrows), numerous mitochondria, rough endoplasmic reticulum profiles, and abundant free ribosomes. (**C**) Regular sarcomeres (Z-disks, A- and I-bands) in the main portion of the fiber; disorganized (chaotic) actin–myosin filaments in the regeneration zones (double parentheses). (**D**) Activated satellite cell (arrows indicate the contact zone with the muscle fiber). (**E**) Schwann cell with a well-developed Golgi apparatus (arrows). (**F**) Regenerative axonal sprouting (arrows). Numerous small axon terminals enclosed by Schwann cell cytoplasm occupy a single synaptic cleft. Scale bars: 25 μm (**A**); 1 μm (**B**,**C**,**E**,**F**); 2 μm (**D**).

In muscle samples subjected to course irradiation (after PBM), signs of muscle tissue regeneration and NMS are clearly visible at the light-optical and ultrastructural levels. In muscle fibers stained according to Van Gieson, chains of centrally located nuclei were observed (Figure 5A). At the ultrastructural level, functionally active myosatellites with large nuclei and a large number of ribosomes and mitochondria were identified (Figure 5D). More often than in control samples, myoblasts with a large number of mitochondria, elements of the rough endoplasmic reticulum, and scattered myofibrils in the process of assembly could be found (Figure 5B). On the NMS side, we found that some terminals were in a state of regenerative sprouting. Several small axon terminals were located within a single synaptic cleft, isolated by Schwann cell cytoplasm (Figure 5F).

### 3.2. Morphometric Analysis of NMS

**Figure 6:**
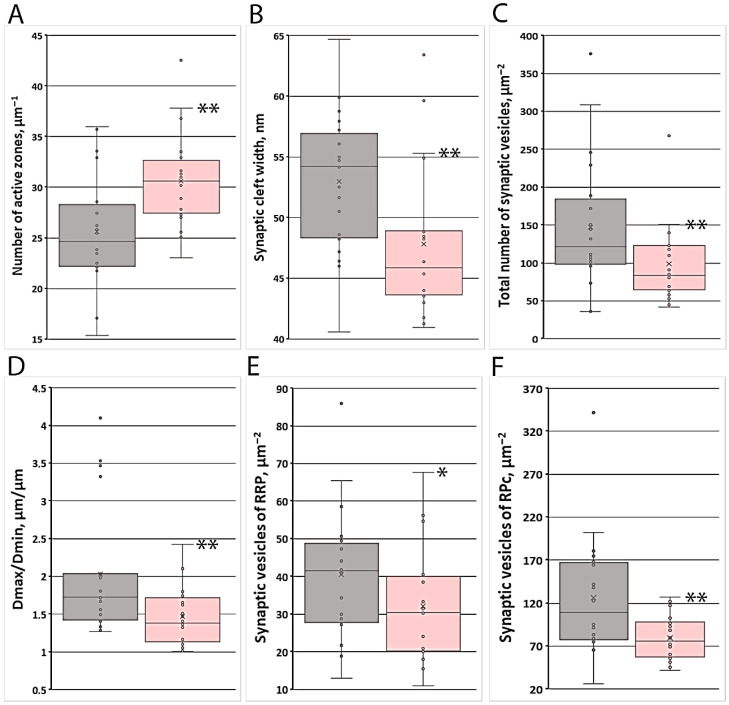
Key morphometric parameters of nerve endings indicating increased functional activity of neuromuscular junctions in aged rats after PBM therapy. Asterisks signify the levels of statistical significance *: *p* ≤ 0.05; **: *p* ≤ 0.01. (**A**) Number of active zones (μm^−1^); (**B**) synaptic cleft width (nm); (**C**) total number of synaptic vesicles (μm^−2^); (**D**) Dmax/Dmin ratio (μm/μm); (**E**) number of synaptic vesicles of the RRP (μm^−2^); (**F**) number of synaptic vesicles of the RPc (μm^−2^). Legend: gray column—control, pink column—PBM.

Morphometric analysis of neuromuscular junctions revealed that animals exposed to photobiomodulation (PBM) exhibited a 12% reduction in the cross-sectional area of nerve endings compared to the control group. The perimeter of the nerve endings showed no significant change. The small diameter of the nerve terminals increased by 14%, whereas the large diameter decreased by 11.7% (Table A2, Appendix A), resulting in a statistically significant 17.6% reduction in the ratio of large to small diameter (*p* < 0.01). The length of the presynaptic membrane remained unchanged, while the length of the postsynaptic membrane increased by 20.1%. Importantly, the number of active zones increased significantly by 23.9% (*p* < 0.01). Consequently, the ratio of the nerve ending perimeter to the length of the postsynaptic membrane (PPT/LPOST) decreased by 29.6%, and the ratio of presynaptic to postsynaptic membrane length (LPRE/LPOST) decreased by 15.4%. In addition, the width of the synaptic cleft was reduced by 15.4% (Figure 6).

Stereological analysis of presynaptic terminals revealed the following changes after photobiomodulation (PBM): the relative volume of axoplasm showed no significant change, whereas the relative volume of synaptic vesicles decreased significantly by 34.4% (*p* < 0.05). The relative volume of mitochondria increased by 19.5%, although this change did not reach statistical significance (*p* > 0.05) (Table A2, Appendix A).

In both control and PBM-treated samples, mitochondria were diffusely distributed throughout the axoplasm, with a predominant concentration in the central region of the nerve terminals. They exhibited round, oval, or elongated shapes. Along with intact mitochondria, organelles with a lightened matrix, fragmented cristae, and disruption of the outer membrane were frequently observed.

Following PBM, the total number of mitochondria per unit area increased by 14.8%, while the average area of individual mitochondria remained virtually unchanged compared to the control group (Table A3, Appendix A). Although the absolute number of defective mitochondria increased due to the overall rise in mitochondrial count, the percentage of organelles with defects in the outer membrane increased by only 7.8%, whereas the percentage of mitochondria with inner membrane defects decreased by 12.1%.

In addition to mitochondria, the presynaptic terminals contained a large number of synaptic vesicles organized into three functional pools: the reserve pool (RP), the recycling pool (RPc), and the readily releasable pool (RRP) (Figure 4F). Morphometric analysis revealed that after photobiomodulation (PBM), the total number of synaptic vesicles per unit area decreased by 34.9% (*p* ≤ 0.05) compared to the control group (Table A4, Appendix A). The number of vesicles in the readily releasable pool (RRP) decreased by 26.7% (*p* ≤ 0.05), and the number in the recycling pool (RPc) decreased by 30.5% (*p* < 0.01). The number of vesicles in the reserve pool (RP) also decreased by 28.3% (*p* > 0.05), although their proportion relative to the total vesicle count increased by 15.4% (*p* > 0.05). Interestingly, the ratio between the vesicle fractions located closer to the presynaptic membrane (within 300 nm) showed no significant change. However, when the number of vesicles in each pool was recalculated relative to the length of the pre- and postsynaptic membranes, statistically significant differences were observed between the control and PBM-treated groups.

## 4. Discussion

In a comprehensive morphological study at the tissue and ultrastructural levels using morphometric methods, we analyzed the functional activity of neuromuscular synapses in old rats before and after PBM. We consider the neuromuscular junction a key component of the “NMS-muscle” functional system according to P.K. Anokhin [26], where the synaptic apparatus ensures adaptive signal transmission from the motor neuron to the muscle fiber by integrating afferent information, central regulation, and peripheral feedback to implement the target motor act. In this system, the NMS is not an isolated element but a dynamic node that determines the effectiveness of muscle contractility, energy supply, and plasticity under age-related changes, which allows us to evaluate the effects of PBM as a systemic response at the level of morphofunctional reorganization.

The results obtained indicate significant changes in the structure of the neuromuscular junction under the influence of PBM in old male Wistar rats. These changes reflect remodeling of the synaptic apparatus aimed at optimizing energy supply, neurotransmitter transmission, and potential regeneration of nerve terminals. Such remodeling is particularly relevant in the context of age-related sarcopenia and NMJ degeneration [27,28,29].

Stereological analysis of nerve ending compartments in experimental samples revealed a reduction in the relative volume of synaptic vesicles and an increase in the relative volume of mitochondria. This change was not accompanied by significant shifts in the relative volume of axoplasm but was combined with the sprouting of nerve terminals, indicating increased neuroplasticity. These effects are consistent with data on the regenerative potential of PBM: in models of sciatic nerve damage in rats, a laser with a wavelength of 660 nm stimulated the growth of axonal processes, improved muscle morphometry, and increased the expression of regeneration genes such as myogenin and calcineurin [30,31]. Regenerative sprouting is likely a manifestation of PBM-induced neuroplasticity, which is particularly relevant for aging animals, in which age-related degenerative processes—such as nerve terminal fragmentation—lead to a 25–30% decrease in muscle strength [30]. These changes also correlate with data on the ability of PBM to stimulate skeletal muscle regeneration by activating signaling pathways, including PI3K/AKT/mTOR, and increasing the expression of muscle regeneration markers [15,32].

Mitochondria are highly plastic organelles that are sensitive to various influences and damage, especially in the tissues of aging animals [33]. In our study, even in control muscle samples, signs of mitochondrial destruction were observed, including swelling, cristae destruction, and membrane damage.

Of particular interest is the increase in the relative volume of mitochondria and their numerical density while maintaining the area of individual organelles in nerve terminals after PBM. These changes indicate PBM-induced mitogenesis without hypertrophy of existing mitochondria. The literature confirms that PBM, especially in the red and near-infrared range, activates cytochrome c oxidase in mitochondria, increasing ATP synthesis and reducing oxidative stress [34,35]. In experiments on the C2C12 cell line, PBM increased mitochondrial membrane potential and ATP synthesis, with a peak response after 3–6 h [36]. Similarly, in models of peripheral nerve damage in rats, PBM (660 nm) increased mitochondrial activity in skeletal muscles, promoting recovery from injury [34].

In our study, after PBM, there was an increase in the proportion of mitochondria with outer membrane defects and a decrease in inner membrane defects, indicating selective mitochondrial remodeling, possibly associated with the activation of mitophagy of damaged outer membrane compartments and restoration of the respiratory chain in the inner membrane. However, PBM had a positive effect on the mitochondria of nerve terminals, contributing to an increase in their relative volume and numerical density. We attribute the increase in the proportion of mitochondria with outer membrane defects to the expansion of their population rather than to the destructive effect of PBM. Membrane defects may be caused by an increased concentration of reactive oxygen species (ROS), which the repair mechanisms, weakened by age-related changes, were unable to fully compensate for.

It is likely that PBM enhances mitophagy of damaged organelles while preserving normally functioning mitochondria, which is consistent with data on the neuroprotective effects of PBM in models of aging and epilepsy, where therapy improved mitochondrial function and cognitive performance [37,38]. In addition, in rodent models of spinal injury, PBM (810 nm) maintained mitochondrial integrity by regulating the balance of mitochondrial division and fusion [39]. These data highlight the potential of PBM to optimize the mitochondrial pool and maintain energy homeostasis in the nerve terminals of aging animals.

Cholinergic neurotransmission is important for the functioning of the central and peripheral nervous systems and is mediated by regulated exocytosis of synaptic vesicles containing acetylcholine (ACh) [40]. Exocytosis, the main mechanism of neurotransmitter release, depends on the dynamic mobilization, recruitment, and replenishment of the vesicle pool in nerve endings—processes that are strictly controlled by membrane proteins [41,42].

In this study, PBM caused significant reorganization of synaptic vesicle pools in neuromuscular junctions. Morphometric analysis showed that after PBM, the total number of synaptic vesicles per unit area in nerve endings decreased. This process affected all three pools to varying degrees. Only the number of vesicles in the ready-to-release pool and the recirculating pool decreased statistically significantly. The number of vesicles in the reserve pool also decreased compared to the control, and their share of the total number of vesicles increased. These results indicate a functional shift in the operation of the synaptic apparatus, which probably enhances the kinetics of initial ACh release and synaptic efficiency in the early stages of stimulation. The decrease in the volume of the reserve pool indicates increased neurotransmitter consumption, which may be accompanied by enhanced synthesis or recycling, consistent with increased NMJ activity [43,44,45].

This mechanism may involve modulation of ion channels under the influence of PBM. Red and near-infrared light (600–1000 nm) is known to affect voltage-gated Ca^2+^ channels and Na^+^/K^+^-ATPase, increasing intracellular Ca^2+^ and promoting vesicle mobilization [46]. This is consistent with reports that PBM (660 nm) increases synaptic density and neuroligin-3 (Nlgn3) expression in neuronal models [47]. At the NMJ, such Ca^2+^-dependent signaling may optimize ACh exocytosis, especially in conditions of age-related synaptic decline characterized by terminal fragmentation and a 25–30% loss of muscle strength [28].

In support of this, it has been shown that PBM regulates nicotinic acetylcholine receptor (nAChR) subunits and improves neuromuscular conduction in models of nerve injury and muscle regeneration in rats [48,49]. Our data extend these observations to aged NMJs, demonstrating that PBM restores the balance of vesicle pools in favor of fast-release compartments, thereby compensating for age-related impairments in synaptic transmission.

The synaptic cleft is the extracellular space of the synapse that separates the presynaptic and postsynaptic membranes. Electron microscopy has revealed that this area contains electron-dense protein material, including cell adhesion molecules, extracellular proteins, and fragments of receptors and ion channels [50]. Aging leads to widening of the synaptic cleft, which impairs acetylcholine uptake and reduces the efficiency of neurotransmitter transmission [51]. In our study, PBM contributed to a reduction in the width of the synaptic cleft in old rats, as confirmed by transmission electron microscopy data. This change probably accelerates the diffusion of acetylcholine to the receptors of the postsynaptic membrane, which is consistent with an increase in the length of the postsynaptic membrane and the number of active zones, indicating the optimization of synaptic transmission under the influence of PBM.

The postsynaptic membrane of animal NMJs underwent expected changes after exposure to PBM. Some authors believe that an increase in the length of the postsynaptic membrane is a negative sign, indicating the compensatory nature of this phenomenon due to weak signal transmission [52], while others note the elongation of the postsynaptic membrane and an increase in its folding as a positive aspect [12]. We tend to consider the elongation of the postsynaptic membrane as a sign of increased functional activity, especially in conjunction with an increase in the number of active zones.

Morphological adaptations of the postsynaptic component of the neuromuscular junction under the influence of PBM in old rats indicate enhanced synaptic function and potential regeneration. The length of the postsynaptic membrane and the number of active zones—key sites of neurotransmitter release—increased, while the width of the synaptic cleft decreased. The length of the presynaptic membrane remained unchanged, indicating asymmetric remodeling in favor of the postsynaptic component. The increase in the number of active sites likely compensates for the decrease in the relative volume of synaptic vesicles, optimizing acetylcholine release and increasing the efficiency of neurotransmitter transmission [47,53]. The narrowing of the synaptic cleft improves the diffusion of acetylcholine across the synaptic space, which may counteract age-related enlargement of the cleft (by 10–20% in rats older than 18 months), leading to a 25–30% decrease in synaptic efficiency and muscle strength [28].

These changes correlate with the regenerative effects of PBM described in models of peripheral nerve damage. For example, PBM with a wavelength of 660 nm accelerated median nerve regeneration in rats by activating the PI3K/Akt signaling pathway, which enhanced the antioxidant response, improved skeletal muscle morphometry (15–20% increase in fiber diameter), and increased nerve conduction velocity [54]. Similarly, PBM (660 nm) increased synaptic density and neuroligin-3 (Nlgn3) expression in neuronal synapses, improving synaptic plasticity in epilepsy models, which may be applicable to NMJs due to similar mechanisms of synaptic protein modulation [47]. Overall, these morphological adaptations—including postsynaptic membrane elongation, an increase in the number of active zones, and synaptic cleft narrowing—contribute to improved motor function, as shown in studies of muscle and nerve regeneration after injury, where PBM (600–1000 nm) stimulated the expression of regeneration markers such as myogenin and improved neuromuscular conduction [55].

The results of our study confirm that PBM with a wavelength of 650 nm and an energy density of 6 J/cm^2^ has a positive effect on body tissues, including the stimulation of regenerative processes [56].

One of the key morphological signs of aging in skeletal musculature is the centering of symplast nuclei in muscle fibers, which is traditionally considered an indicator of degenerative processes. However, according to the research by Sayed et al. (2016), the migration of nuclei to the center of the fiber may serve as a signal for the activation of myosatellite cells, contributing to the regeneration of muscle tissue [6,57]. In our study, a similar pattern was observed: in the limbs subjected to PBM, there was an increase in the activity of myosatellite cells, confirmed at the level of electron microscopy, which indicates the potential involvement of PBM in stimulating regenerative processes in aging muscle tissue.

Electron microscopy revealed in the skeletal muscles (*m. vastus lateralis*) of aged rats the presence of myoblasts containing a large number of mitochondria, elements of the rough sarcoplasmic reticulum, and myofibrils in the process of assembly. Also observed were activated myosatellitocytes with large nuclei and increased content of ribosomes and mitochondria. These changes indicate the activation of myosatellite cells, which play a key role in the regeneration of muscle tissue, especially in conditions of age-related sarcopenia, characterized by a decrease in muscle mass and strength by 25–30% [28].

Age-related changes in neuromuscular junctions, such as mitochondrial dysfunction and synaptic vesicle deficiency, have similarities with sarcopenia in humans [12,14,58]. Contralateral control minimized individual variability; however, systemic effects of PBM, mediated by circulating factors (e.g., cytokines or growth factors), cannot be completely excluded, since PBM can modulate redox signaling and mitochondrial activity systemically [35]. High-resolution respirometry could confirm functional correlates, such as enhancement of oxidative phosphorylation; however, in this study, the emphasis was placed on morphological analysis [59]. The limitations of the study include the absence of long-term observation of PBM effects and molecular data, such as ATP levels or expression of mitochondrial biogenesis genes (e.g., PGC-1α), which could clarify the mechanisms of the observed changes [35]. We explicitly note that direct functional validation and comparative studies with young animals are required to establish therapeutic efficacy.

## 5. Conclusions

In conclusion, photobiomodulation induces significant ultrastructural changes in the neuromuscular junctions of aged rats, including an increased number of active zones, a longer postsynaptic membrane, a narrower synaptic cleft, and mitochondrial hyperplasia. These morphological alterations indicate enhanced synaptic efficiency and suggest a potential regenerative effect of PBM on age-related neuromuscular degeneration. Future studies combining structural and functional assessments are warranted to confirm the translational potential of these findings.

## Data Availability

The original results presented in the study are included in the article. The primary quantitative data are publicly available on Zenodo at https://doi.org/10.5281/zenodo.19220218. All data and the original code generated for analysis will be made available upon reasonable request.

## Abbreviations

The following abbreviations used in this manuscript:

ACh	Acetylcholine
AM	Cross-section area of mitochondrion
APT	Presynaptic terminal area
DMAX	Maximum presynaptic terminal diameter
DMIN	Minimum presynaptic terminal diameter
LPRE	Length of the presynaptic membrane
LPOST	Length of the postsynaptic membrane
NAZ	Number of active zones
NMS	Neuromuscular synapse
PBM	Photobiomodulation
PPT	Presynaptic terminal perimeter
RRP	Readily releasable pool (of synaptic vesicles)
RP	Reserve pool (of synaptic vesicles)
RPc	Recycling pool (of synaptic vesicles)
TEM	Transmission electron microscopy
WSC	Width of the synaptic cleft

## Appendix A

**Table A1:** Primary morphometric parameters of the NMS analyzed in the study.

Parameter	Symbol	Control	PBM
		*n*	*n*
Presynaptic terminal area (µm^2^)	A_PT_	20	20
Presynaptic terminal perimeter (µm)	P_PT_	20	20
Maximum presynaptic terminal diameter (µm)	D_MAX_	20	20
Minimum presynaptic terminal diameter (µm)	D_MIN_	20	20
Presynaptic membrane length (µm)	L_PRE_	20	20
Postsynaptic membrane length (µm)	L_POST_	20	20
Synaptic cleft width (nm)	W_SC_	340	340
Number of active zone (number)	N_AZ_	224	261
Cross-section area of mitochondrion (µm^2^)	A_M_	140	136
Total number of mitochondria (number)	N_M_	140	136
Mitochondrial number with inner membrane defects (number)	N_IMD_	49	62
Mitochondrial number with outer membrane defects (number)	N_OMD_	41	41
Number of Readily Releasable Vesicles (number)	N_RRP_	307	210
Number of Recycling Vesicles (number)	N_RPC_	986	575
Number of Reserve Vesicles (number)	N_RV_	1657	1189

**Table A2:** Electron Microscopic Morphometric Analysis of Presynaptic terminal and Neuromuscular Junctions in *m. Vastus Lateralis* of Aged Rats: Effects of Photobiomodulation.

Morphometric Parameters	Control		PBM		Δ%
Presynaptic terminal area, µm^2^	4.24	[2.59–5.51]	3.73	[2.41–5.04]	−12.0
Presynaptic terminal perimeter (P_PT_), µm	8.20	[6.42–10.22]	8.08	[6.35–8.61]	−1.5
Maximum presynaptic terminal diameter (D_MAX_), µm	3.00	[2.17–3.66]	2.65	[1.96–3.11]	−11.7
Minimum presynaptic terminal diameter (D_MIN_), µm	1.57	[1.37–1.81]	1.79	[1.47–2.12]	+14.0
D_MAX_/D_MIN,_ µm/µm	1.73	[1.47–2.04]	1.39	[1.15–1.68] **	−17.6
Presynaptic membrane length (L_PRE_), µm	4.12	[3.77–5.30]	4.13	[3.47–5.25]	+0.2
Postsynaptic membrane length (L_POST_), µm	18.14	[12.53–23.52]	21.79	[12.93–26.71]	+20.1
P_PT_/L_POST,_ µm/µm	0.54	[0.32–0.69]	0.38	[0.34–0.45]	−29.6
L_PRE_/L_POST,_ µm/µm	0.26	[0.18–0.38]	0.22	[0.20–0.26]	−15.4
Number of active zones per unit L_PRE,_ µm^−1^	24.7	[22.2–27.7]	30.6	[27.7–32.0] **	+23.9
Synaptic cleft width, nm	54.2	[48.5–56.4]	45.9	[43.9–48.9] **	−15.3
Axoplasm volume fraction, %	80.4	[72.1–85.3]	79.5	[73.5–83.4]	−1.1
Mitochondrial volume fraction, %	12.8	[7.2–23.7]	15.3	[12.1–22.0]	+19.5
Synaptic vesicle volume fraction, %	6.4	[4.2–7.0]	4.2	[3.6–4.9] *	−34.4

Data are median [25th percentile–75th percentile]. * *p* ≤ 0.05; ** *p* ≤ 0.01.

**Table A3:** Electron Microscopic Morphometric Analysis of Mitochondria in Presynaptic terminal NMJ of *m. Vastus Lateralis* from Aged Rats: Effects of Photobiomodulation.

Morphometric Parameters	Control		PBM		Δ%
Cross-section area, µm^2^	0.095	[0.082–0.116]	0.093	[0.084–0.128]	−2.1
Total number, µm^−2^	13.5	[10.5–22.4]	15.5	[13.2–19.8]	+14.8
Number with inner membrane defects, µm^−2^	6.5	[2.0–7.7]	7.7	[4.7–11.0]	+18.5
Number with outer membrane defects, µm^−2^	4.1	[3.1–7.1]	4.9	[4.0–6.5]	+19.5
Fraction with inner membrane defects, %	41.4	[19.2–50.0]	36.4	[33.3–58.2]	−12.1
Fraction with outer membrane defects, %	30.9	[20.0–50.0]	33.3	[25.8–44.6]	+7.8

Data are median [25th percentile–75th percentile].

**Table A4:** Electron Microscopic Morphometric Analysis of Synaptic Vesicles in Motor Endplates of *m. Vastus Lateralis* from Aged Rats: Effects of Photobiomodulation.

Morphometric Parameters	Control		PBM		Δ%
Number of synaptic vesicles per unit area (µm^−2^)					
Total number	375.5	[246.2–411.2]	244.4	[210.9–289.5] *	−34.9
RRP	41.6	[28.5–47.8]	30.5	[20.6–39.1] *	−26.7
RP_C_	109.3	[78.3–165.1]	76.0	[59.2–97.1] **	−30.5
RP	181.7	[124.9–240.9]	130.2	[110.0–155.2]	−28.3
Number of synaptic vesicles per unit presynaptic membrane length (µm^−1^)					
RRP	33.1	[25.3–39.6]	27.2	[21.9–29.7] **	−17.8
RP_C_	9.1	[6.7–12.1]	5.2	[3.9–7.3] **	−42.9
RP	102.7	[76.4–139.2]	66.4	[50.9–79.1] **	−35.3
Number of synaptic vesicles per unit postsynaptic membrane length (µm^−1^)					
RRP	28.5	[18.0–39.3]	13.9	[11.1–17.9] **	−51.2
RP_C_	146.9	[95.0–237.1]	117.2	[77.5–156.8]	−20.2
RP	47.3	[23.1–75.1]	24.3	[18.3–35.6] *	−48.6
Fraction of Pool (%)					
RRP	11.8	[7.0–16.9]	12.0	[6.5–17.0]	+1.7
RP_C_	32.4	[29.9–40.6]	30.3	[26.7–35.3]	−6.5
RP	50.0	[45.9–63.0]	57.7	[48.0–60.2]	+15.4

Data are median [25th percentile–75th percentile]; ****** *p* ≤ 0.01; ***** *p* ≤ 0.05. RRP—Readily Releasable Pool; RP_C_—Recycling Pool, RP—Reserve Pool.
